# Consequences of Poverty on Economic Decision-Making: Assessing the Verisimilitude of the Cognitive Mechanism

**DOI:** 10.3389/fpsyg.2020.00171

**Published:** 2020-02-13

**Authors:** Matúš Adamkovič

**Affiliations:** Institute of Psychology, Faculty of Arts, University of Prešov, Prešov, Slovakia

**Keywords:** economic situation, poverty, cognitive mechanism, economic decision-making, poverty perpetuation

## Abstract

The paper aims to assess the verisimilitude of the hypothesized model of poverty perpetuation which links socioeconomic situation and economic preferences via cognitive load, executive functions, and intuitive/deliberative decision-making styles. In order to test the model against the data, three studies (exploratory, confirmatory, and replication) were conducted with a total sample size of 1182 participants. The results showed that neither the proposed model as a whole found the required support in the data nor the consequent, theoretically justifiable, respecifications improved its fit so that it could be deemed acceptable. Simultaneously, the dyadic relationships between the variables were mainly found to be weak. The sensitivity analysis revealed that the majority of the observed estimates varied substantively depending on the arbitrary analytic decisions of the researcher. In summary, the hypothesized cognitive mechanism does not explain what economic decision-making depends on nor why people fall into poverty traps. The paper discusses several plausible sources of the negative findings and possible directions for future research are suggested.

## Introduction

Poverty is one of the greatest challenges facing society, persisting in spite of efforts to alleviate it. Besides the well-known cultural, social, environmental, and health-related causes, the inability to escape from poverty might be determined by psychological factors which can be studied at the level of individuals. To a certain extent, a person’s financial situation is determined by the economic decisions that they make. However, it is unclear what drives these decisions and whether (and how) they are determined by a person’s actual economic situation.

People living in poverty are said to have suboptimal economic preferences that could play an important part in poverty perpetuation (Kraay and McKenzie, 2014). In particular, their willingness to delay gratification is claimed to be lower (see, for example, Griskevicius et al., 2011; Brown et al., 2015) and they tend to be more reluctant to take risks when a reward is involved (Haushofer and Fehr, 2014). Yet, it would be an inappropriately simplified assumption to say that such decisions are directly caused by the conditions of poverty *per se*. Previous research has shown that poverty is related to factors like stress (Haushofer and Fehr, 2014), cognitive load (Schilbach et al., 2016), worsened cognitive functions (Mani et al., 2013), and present-oriented behavior (Griskevicius et al., 2011). These, as well as the other correlates of poverty (for a review, see Pepper and Nettle, 2017), are often identical to the determinants of decision-making and preferences (see, for example, Burks et al., 2009; Capraro, 2019). If these variables are indeed related, it should be possible to derive a tenable model of a mechanism that could explain why people cycle themselves in their financial situation. Until recently, there had been no formal model dedicated to grasping poverty perpetuation from the perspective of an individual. Therefore, Adamkovič (2019) proposed a theoretical model explaining how poverty influences a person’s economic decision-making. The model has linked socioeconomic status and economic preferences via cognitive load, executive functions, and intuitive/deliberative styles of decision-making. While the authors provide a narrative review and psychological rationale of the causal mechanism, the model has not yet been tested against data. The current study is an empirical follow-up to this narrative review. In this paper, an additional theoretical background to the model will firstly be provided, followed by testing the model against three datasets and consequently discussing the findings.

### The Model and Its Caveats

This model depicts poverty as creating cognitive load in the form of experiencing negative affect and stress (Adamkovič, 2019). The cognitive load, not poverty *per se*, impairs a person’s executive functions such as attention focus, working memory, and self-control capacity. The executive functions are mutually related, but only self-control capacity has a direct effect on a person’s intuitive/deliberative style of thinking. Both self-control and the intuitive/deliberative style of thinking causally affect economic decision-making. In the presented model, economic decision-making is represented by time-discounting and risk preference when reward/loss is involved. The economic preferences are also determined by one’s financial literacy.

The initially proposed model has several caveats that should be addressed. Some of them have already been taken into account in the previous paper, although more explicit clarification is needed. (1) Firstly, it disregards the fact that poverty is a multidimensional construct (Smeeding, 2015) and that its subjective assessment likely plays a more important role in the executive processes than its objective indicators (Mani et al., 2013). (2) Secondly, while the term poverty is used, a whole range of economic status is referred to in the paper. Indeed, focusing only on people who are considered poor (whatever operationalization is used) will lead to an unwanted phenomenon called range restriction (see, Pedhazur and Schmelkin, 1991). If the sample was restricted to the poor, it would lead to a loss of information (e.g., if the model holds true, it would be easy to claim that the result was unique for the poor even though the process could be same for the whole spectrum of economic situations) and likely attenuated effect sizes. (3) The fundamental notion upon which the model has been built is open to question. The claim that poverty leads to suboptimal economic trade-offs does not have as strong support in the evidence as the narrative often presents. For instance, in a large cross-national study, the relationship between household income and time-discounting and risk-taking is very weak at β = 0.04 and β = 0.06, respectively, after controlling for covariates (Falk et al., 2015). Similarly, weak associations can be found throughout the relevant literature (for an overview, see Adamkovič et al., 2018). This is with the exception of Brown et al. (2015) who found that people with liquidity constraints are about 20% more likely to choose a more present-oriented option. (4) In terms of economic preferences, it is necessary to note that the model captures only the subset, intertemporal, and risk preferences that are related to financial decisions. Although this choice picks up on the largest Global Preference Survey (Falk et al., 2015), there are other types of financial behavior and preferences (e.g., social preferences; Fehr and Schmidt, 1999) that can play an important role in the model of poverty perpetuation. These alternatives will be briefly discussed as possible directions for future research. For simplicity, intertemporal and risk preferences are labeled by the superior term, economic preferences, throughout the paper. (5) Another potential pitfall of the model is the issue of modeling causality from cross-sectional data in behavioral sciences (see, for example, Pearl, 2009; Rohrer, 2018). Although the authors claim that the model describes a potential cognitive mechanism of poverty perpetuation, it is acyclic (as a cyclic model of this complexity would not meet the assumptions of order condition and rank condition; Kline, 2016) and the feedback loop from economic decision-making to poverty causing the poverty trap is only theoretically assumed. A further potential causality-related problem is that conditioning on colliders or mediators leads to biased estimates (Greenland, 2003; Pearl, 2009). However, the model implicitly deals with these issues and only a slight modification (described in the following section) has been made in order to have the causality of the paths as theoretically justifiable as possible. (6) In addition to these points, omitted-variable bias is another potential issue. One could argue that certain key elements are missing from the model, such as intelligence, perceived reliability of the environment, motivational factors, macroeconomic and political expectations, and sociodemographic variables (e.g., gender, time spent living in poverty, social unit, etc.). While this may be true, all statistical models are a simplification of real-world processes and cannot comprise all possible variables. Having and testing alternative models (or a similar model with different choices throughout the process – see researcher degrees of freedom; Simmons et al., 2011; Wicherts et al., 2016) is a subject of constructive and conceptual replications. (7) One of the core assumptions of the model is the exhaustion of one’s mental resources. This notion is usually associated with the ego-depletion theory, which has been falsified in the majority of replication studies and meta-analyses. However, a recent review of the literature concluded that the evidence for/against the ego-depletion theory is inconclusive and that the phenomenon might exist in specific conditions (Friese et al., 2019). Nonetheless, there remains an implication that the proposed model might not hold true. While the concept of ego-depletion is popular, it is not the only theory which depicts how (the lack of) self-control determines one’s decision-making. There are two competing models of self-control: the Process model (Inzlicht and Schmeichel, 2012) and the CoMo model (Dang, 2018). In particular, the CoMo model can explain the motivational shifts that lead an individual toward making more hedonistic, or in this case more myopic, choices.

### The Current Version of the Model

**FIGURE 1:**
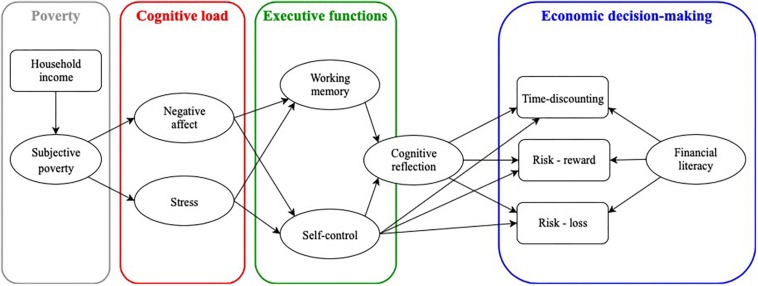
Conceptual visualization of the hypothesized cognitive mechanism of poverty perpetuation.

In order to verify the verisimilitude of the proposed cognitive mechanism of poverty perpetuation, the variables and their relationships were modeled as closely to the initial model as possible (although slight modifications have been made). The latent variable representing poverty has been split into two components – the objective economic situation and subjective economic situation. The objective economic situation has a direct effect on its subjective perception but does not causally affect any other variable in the model. In other words, the subjective perception of one’s own economic situation blocks the causal flow from the objective economic situation to experiencing negative affect and stress (i.e., from the perspective of objective economic indicators, a person can be considered poor but it is her perception of the situation, not the wealth itself, that affects her levels of negative affect and distress). For practical reasons associated with the data collection, the variable representing one’s attention had to be omitted from the model. Furthermore, given the current evidence that shows negligible relationships between working memory and self-control (Nêcka et al., 2018; Singh and Göritz, 2018), it was refrained from the initial assumption that the two are related. The conceptual visualization of the current version of the model is depicted in Figure 1. The subsequent testing of the model against the data will reveal whether this narrative-based cognitive mechanism can indeed explain the process of poverty perpetuation.

## Materials and Methods

### General Information

The aim of the paper is to verify whether the hypothesized model depicting how poverty influences one’s economic decision-making via cognitive load, executive functions, and intuitive/deliberative styles of thinking holds true when confronted with data. It also aims to examine the magnitude of the relationships between the variables in the model. In order to do this, three studies were conducted: exploratory, confirmatory, and replication. In the case that the tested model would be falsified in the exploratory dataset, the only acceptable modifications would be those that are theoretically justifiable and still fit the original theoretical framework of the proposed mechanism. The modified model would then be cross-validated on the confirmatory dataset. The third dataset would be used for a constructive/conceptual replication (see Hüffmeier et al., 2016) of the findings.

Two sets of data (*N*_1_ = 430, *N*_2_ = 500) were initially collected to answer the research question. Due to financial constraints, the participants were incentivized in the form of a small non-financial reward. In order to see how the results would change when participants were incentivized with real money, a third research sample (*N*_3_ = 252) was added into the analyses. The data for the third wave of data collection came from an unpublished experiment (see its preregistration)^1^. The data might be biased because of the experimental situation (note that the experimental conditions had no effect on the outcome variable – time-discounting; also note that the experimental situation was administered just before the time-discounting tasks, after the rest of the test battery had been completed). Therefore, these results are reported as a piece of additional evidence and the specific estimates should be interpreted with particular caution. This study is also not considered as a close replication but rather as an attempt of a constructive/conceptual replication of the findings. Some of the measures slightly differ (e.g., the financial satisfaction scale consisted of three items instead of six) and it does not include negative affect and risk preference variables.

### Participants, Data Collection, Data Screening and Power Analysis

The data were collected in three waves: (1) *N*_1_ = 430 (218 women), Age_1_ = 40.05 ± 11.88 years; (2) *N*_2_ = 500 (250 women), Age_2_ = 39.57 ± 11.48 years; (3) *N*_3_ = 252 (204 women), Age_3_ = 34.92 ± 0.31 years. The data from the first two samples were collected online via a local agency specializing in data collection and market research. The agency works on principles similar to MTurk. In these cases, the participants were rewarded with credits that they could spend on a variety of products offered by the agency. In the third data collection, participants were recruited in person and the materials were administered in the paper-pencil form. This time, the participants were incentivized with a real monetary reward ranging between 6 and 12 euros, depending on their intertemporal preference. All the data collection methods were ethically approved by the Ethics Board at the Institute of Psychology, University of Prešov. All the data were collected as part of a bigger data collection for the research project “Psychological causes and consequences of poverty” (grant no. APVV-1504-04). Every data collection lasted about 30 min on average, per participant.

At the time of the data collection, 63% of the total number of participants were employed or self-employed, 11% were unemployed, 7% considered themselves as primarily full-time students, and 10% were retired or receiving other forms of pension (e.g., disability support pension) benefits. The remaining 9% found the option “other” as the most suitable. 2% of the sample had achieved a maximum of primary education, 60% had finished their secondary education and the remaining 38% had successfully achieved an academic degree.

The datasets were screened for careless respondents. In the online data collections, we examined the long strings (see Curran, 2016; the participants with long string length >2.5 SD were considered to be potentially careless) and participants’ answers to open-ended questions. In the case of the face-to-face recruitment, the long strings were also screened (based on the same rules as used in the previous cases) and checked whether or not they passed the attention checks. If a participant failed more than 1 out of 3 of the attention checks, they were considered to be careless. A total sample of 1143 participants (*N*_1_ = 418, *N*_2_ = 485, *N*_3_ = 240) remained after excluding the seemingly careless respondents.

All the sample sizes were determined by the budget available for data collections. A RMSEA-based power analysis (α = 0.05; H_*a*_ RMSEA = 0.08; H_0_ RMSEA = 0.04; df > 200 given the approximate smallest number of the estimated relationships in the model) yielded more than 99% power to indicate that the whole structural model is misspecified. Even the research design with the smallest number of participants (*N*_3_ = 240) showed almost 90% power (given the conventional α = 0.05 for the two-sided test) to be able to detect the smallest effect size of interest, *r* = 0.2, for the estimated relationships.

### Measures

The participants were administered a test battery composed of several measures. A brief summary of the measures is provided, including their reliabilities (McDonald’s omega total coefficient) estimated on the samples. A more detailed overview of the employed measures can be found at https://osf.io/z6sej/. The descriptive statistics are shown in Table 1.

**TABLE 1:** Descriptive statistics.

	**Study 1**				**Study 2**				**Study 3**			
	**M**	**SD**	**ω**	***n***	**M**	**SD**	**ω**	***n***	**M**	**SD**	**ω**	***n***
Income	561	295	−	1	606	328	−	1	644	369	−	1
SES	5.30	1.69	−	1	4.75	1.60	−	1	−	−	−	−
ECO	2.92	0.78	0.92	6	2.87	0.79	0.93	6	4.66	1.72	0.79	3
Wealth	4.64	1.41	−	1	4.66	1.45	−	1	−	−	−	−
NEG	2.71	0.66	0.90	10	2.59	0.68	0.90	10	−	−	−	−
Stress	1.83	0.54	0.84	10	2.18	0.58	0.86	10	2.49	0.65	0.81	10
WM	8.75	1.57	0.81	10	5.70	2.7	0.86	9	3.35	1.85	0.83	8
SC	3.31	0.55	0.81	13	3.33	0.52	0.79	13	3.41	0.56	0.75	13
CR	2.47	1.78	0.87	6	2.55	1.79	0.88	6	2.10	1.41	0.80	6
TD 1	18.23	10.70	−	5	17.45	10.91	−	5	11.22	7.77	−	5
TD 2	−	−	−	−	0.03	0.06	−	27	−	−	−	−
TD 3	−	−	−	−	−	−	−	−	2.42	1.45	−	1
Risk−R	8.66	7.48	−	5	8.75	6.74	−	5	−	−	−	−
Risk−L	21.56	7.70	−	5	22.2	7.00	−	5	−	−	−	−
FL	3.75	1.60	0.81	6	4.29	1.88	0.78	6	3.62	1.58	0.70	8

*Income, equivalized household income; SES, subjective perception of SES (range 1−10; a higher score indicates a higher SES); ECO, financial satisfaction scale (range 1−5 for Study 1 and 2, range 1−10 for Study 3; a higher score indicates higher satisfaction with one’s own economic situation); Wealth, perception of own poverty/wealth (range 1−9; a higher score indicates more wealth); NEG, negative affect (range 1−5; a higher score indicates a higher level of negative affect); Stress, perceived stress (range 0−4; a higher score indicates higher stress); WM, working memory (scale 0 – 10/9/8; a higher score indicates better working memory); SC, self-control (range 1−5; a higher score indicates higher self-control); CR, cognitive reflection (scale 0−6; a higher score indicates more deliberative thinking); TD 1, staircase model of time-discounting (range 1−32; a higher number indicates higher time-discounting); TD 2, IMCQ measure of time-discounting (number indicates the rate of discounting); TD 3, time-discounting with a real monetary reward (ordered categorical; range 1−4; a higher number indicates higher time-discounting); Risk-R, staircase model of risk preference when a reward is involved (range 1−32; a higher number indicates higher risk preference); Risk-L, staircase model of risk preference when a loss is involved (range 1−32; a higher number indicates higher risk preference); FL, financial literacy (scale 0−6/8; a higher score indicates higher financial literacy).*

Objective poverty (objective economic situation) was measured as equivalized household income (Hagenaars et al., 1994). Subjective poverty (subjective perception of one’s own economic situation) was reflected by three indicators. These were the MacArthur scale of subjective social status (Adler and Stewart, 2007; Giatti et al., 2012), the mean score of the created 6-item scale of financial satisfaction (ω = 0.92–0.93), and the 1-item perception of own poverty/wealth. The only exception was in Study 3 when just three items of the financial satisfaction scale were administered (ω = 0.92). Negative affect was assessed by PANAS (Watson et al., 1988; ω = 0.92) and the level of perceived stress was measured by the Perceived Stress Scale (Cohen et al., 1983, ω = 0.81–0.86). A digit span test was used to measure working-memory (ω = 0.81–0.86). In order to measure participants‘ self-control, the Self-control scale (Tangney et al., 2004, ω = 0.75–0.81) was used. A test of cognitive reflection containing 3 items from the original Cognitive Reflection Test (Frederick, 2005) and 3 additional items (Oldrati et al., 2016) were used to examine the tendency of intuitive/deliberative thinking (ω = 0.80–0.87). The Staircase time and risk modules were used to assess participants’ economic preferences (Falk et al., 2016). In the case of risk preference involving a loss, the Staircase module was used, and reduced the amount of money to half. In Study 2, IMCQ (Kirby et al., 1999; Kaplan et al., 2016) was also administered as an additional measure of time-discounting. In Study 3, time-discounting was assessed as the participant preference between 6 euros now, 8 euros in 10 days, 10 euros in 20 days, and 12 euros in a month. Financial literacy was measured using 6/8 items proposed by Lusardi (2008; ω = 0.70–0.81).

### Statistical Analysis

Firstly, all datasets were screened for careless responses and improbable values. Then the descriptive statistics were both statistically and visually checked. It was assumed that there were no outliers as it is believed that these data points represent the true distribution of the construct in the population. The data were not transformed, except for dividing equivalized household income by 100 to reduce its variance and allow for easier convergence when estimating the models. No missing data were present in the two online data collections. In Study 3, approximately 8% of the data were missing and were imputed using the mice package (van Buuren and Groothuis-Oudshoorn, 2011).

The data from Study 1 was used to examine the factor structures of the measured constructs (measurement models). Items with factor loadings <0.40 were omitted from further analyses (see Stevens, 1996). Other items were excluded based on a combination of high cross-loadings, residuals, and modification indices. Parcels from the remaining items were then created to reduce the number of indicators (note: other approaches were also applied to estimate the measurement models. For more details, see section “Sensitivity Analysis”). This dataset was also used to examine and address any other misspecifications in the hypothesized model. However, this was only in the case that the modifications were theoretically justifiable and did not substantially diverge from the proposed mechanism. The original model, as well as the re-specified ones, were then (dis)confirmed on the data from Study 2. Study 3 served to see how the results would replicate under slightly different circumstances (i.e., the method of data collection, slightly different measures, omitted variables of negative affect and risk preferences, or addition of a real monetary reward).

The structural models were estimated in R package lavaan (Rosseel, 2012). The models were estimated using WLSMV (means- and variance-adjusted weighted least squares) with all the variables (except for the equivalized household income) being modeled as ordinal. The models were regarded as falsified based on their chi-square value (a significant chi-square test statistic indicates a misfit of the model) as it is the only formal omnibus test of the (mis)fit of the whole model (Ropovik, 2015). In the exploratory part, the model was adjusted in order to achieve a non-significant chi-square, but only as far as the modifications were theoretically justifiable and in accordance with the main idea behind the proposed mechanism. The fit of each model was diagnosed employing the scaled conventional approximate fit indices (AFI), namely, CFI, TLI, RMSEA, and SRMR.

In addition to the frequentist approach of estimating the paths, the degree of comparative evidence was also assessed calculating the approximate Bayes Factors (BF). BFs represent the relative evidence of the data supporting the alternative hypothesis (the parameter is freely estimated) over the null (the parameter fixed to 0). Furthermore, the posterior probability was estimated – the probability of the observed estimates being non-zero, assuming the equal prior odds (1:1) of H_0_ and H_*a*_ being true.

The code and data are available at: https://osf.io/qy8mn/.

### Sensitivity Analysis

As the results could be subjected to different analytical decisions when analyzing the data, it was decided to inspect their robustness. In particular, the focus was on examining how different approaches to the construction of measurement models shape the obtained estimates, as the measurement models of psychological variables could often be unstable. Therefore, the same structural model was computed multiple times using the different types of measurement models, such as: (1) item parceling; (2) using the exploratory CFA-based items; (3) using all items; and (4) using only the items that best represent the latent construct (see Hayduk and Littvay, 2012), whilst taking into account a combination of their statistical parameters and subjective assessment of their content validity (for all CFAs estimates see Supplementary Table 1). This sort of sensitivity analysis does not cover all the possible choices made by the researchers, although it addresses the majority of the arbitrary analytic choices that could have had a substantial impact on the results. A brief summary of the sensitivity analysis is presented in the results and interpreted in the discussion.

## Results

### Study 1 – Exploratory Testing of the Model

Before testing the actual model against the data, the factor structure of the measures was examined via CFA. The measurement models were then modified based on the previously described criteria. There were three items (no. 3, 5, and 7) which were omitted from the PANAS, four items (no. 1, 4, 5, and 7) from the PSS, the first 3 items from the digit span test, and five items (no. 1, 2, 6, 8, and 12) from the Self-control scale. The Cognitive Reflection Test, as well as the Financial Literacy Scale, remained intact. These modifications to the measurement models led to a substantial increase in their fit^2^. Despite these changes, the significant chi-square statistics of the modified PANAS, PSS, and Self-control scale still indicated a misspecification of the factors. In order to preserve the content validity of the scales, no further modifications/exclusions were made to forcefully obtain a non-significant chi-square value. The reported results were primarily obtained from the measurement models formed by parceling the remaining items.

The obtained estimates indicate that the hypothesized model (see Figure 1 for a conceptual visualization) substantially deviates from the observed data [χ^2^(78) = 839.76, *p* < 0.001; CFI = 0.76; TLI = 0.82; RMSEA = 0.15, 95% CI (0.15,0.16); SRMR = 0.11]. After a thorough inspection of the residual matrix, modification indices, and zero-order bivariate correlations, it was decided that the only theoretically justifiable adjustment to the model would be to add a covariance term between negative affect and stress. This led to a decrease in the chi-square statistic to half its original value. Nonetheless, it still indicated a misspecification in the model [χ^2^(80) = 422.02, *p* < 0.001]. The potential reasons for the observed misfit were examined again. The modification indices suggested adding a covariance between cognitive reflection and financial literacy, or a reversed causality between economic preferences and financial literacy (in a way that economic preferences determine one’s financial literacy). The other observed misspecifications were either negligible from a statistical perspective, or hardly justifiable. Due to problems with financial literacy, it was decided to exclude it from the model in the exploratory manner. With financial literacy excluded, the approximate fit indices showed values that are consensually considered very good – CFI = 0.97; TLI = 0.98; RMSEA = 0.06, 95% CI [0.05, 0.09]; SRMR = 0.05. However, the omnibus chi-square test still indicated the model‘s misfit [χ^2^(74) = 188.78, *p* < 0.001]. No further modifications were made as there was no appropriate theory to support them.

### Study 2 – Confirmatory Testing of the Model

Since the measures of working memory and financial literacy differed from Study 1, the CFAs were computed. In a similar way to the exploratory testing, the first 3 (easiest) items of the digit span test were excluded. One newly added item from the Financial Literacy Scale was also excluded because it did not load on the latent factor at all. The modified scales yielded seemingly very good AFI, but the chi-square test revealed beyond-chance deviations from the data (please note that the results of the CFAs for all 3 studies are available at: https://osf.io/ma9pu/).

Study 2 showed very similar results to those from Study 1 when the proposed model was tested against the data [χ^2^(83) = 1024.33, *p* < 0.001; CFI = 0.77; TLI = 0.81; RMSEA = 0.15, 95% CI (0.15, 0.16); SRMR = 0.11]. In respect to the exploratory testing, a covariance was allowed between negative affect and financial literacy. This again led to an increase in model fit, particularly reducing the chi-square value to half [χ^2^(83) = 546.62, *p* < 0.001]. However, the model can still be considered falsified. Given the issues with financial literacy in the previous data analysis, it was excluded from the model to see whether the results would replicate or not. The fit parameters of the model without financial literacy were very similar to the estimates observed in Study 1. The maximum difference between AFIs was equal to ± 0.03.

### Study 3 – Conceptual Replication

All the measures in Study 3 - apart from the shortened version of the financial satisfaction scale – had already been administered in the previous studies, and hence their modified forms were used. The whole model demonstrated a rather poor fit [χ^2^(67.20) = 213.52, *p* < 0.001; CFI = 0.76; TLI = 0.78; RMSEA = 0.10, 95% CI (0.09, 0.11); SRMR = 0.10]. Since Study 3 did not include the negative affect measure, the only potential modification corresponding to the previous findings was to remove financial literacy. The exclusion of financial literacy led to a slightly better fit [Δχ^2^(12.34) = 41.63, *p* < 0.001]. After a closer examination of the model‘s misfit, it was found that excluding cognitive reflection from the model would be the main modification that would substantially improve its parameters. Since this potential adjustment goes against the theoretical framework of the model, this possibility was not investigated further, although it is addressed in the discussion. This replication has shown that the proposed model does not fit the data, even using real financial incentives. Table 2 shows a summary of the chi-square statistics and AFIs in all the estimated structural models.

**TABLE 2:** Model fit parameters of the tested models for all 3 studies.

**Model**	**χ^2^**	**df**	**p**	**CFI**	**TLI**	**RMSEA (95% CI)**	**SRMR**
**Study 1**							
Initial	839.76	78	<0.001	0.76	0.82	0.15 [0.15, 0.16]	0.11
W/cov.	422.02	80	<0.001	0.90	0.92	0.10 [0.10, 0.11]	0.09
W/o FL	188.78	74	<0.001	0.97	0.98	0.06 [0.05, 0.09]	0.05
**Study 2**							
Initial	1024.33	83	<0.001	0.77	0.81	0.15 [0.15, 0.16]	0.11
W/cov.	546.62	83	<0.001	0.89	0.91	0.11 [0.10, 0.11]	0.09
W/o FL	311.47	73	<0.001	0.94	0.96	0.08 [0.08, 0.09]	0.06
**Study 3**							
Initial	231.52	67.20	<0.001	0.76	0.78	0.10 [0.09, 0.11]	0.10
W/o FL	171.89	54.86	<0.001	0.81	0.83	0.09 [0.08, 0.11]	0.09

*Initial, the initial conceptualization of the model; W/cov., the model with a covariance term between negative affect and stress; W/o FL, the model without financial literacy.*

### Regression Estimates and Their Stability

Over 50 regression coefficients have been estimated from the primarily reported analysis, and thus will not be interpreted and discussed separately. The exact values of the coefficients for the model with the covariance between negative affect and stress (applies for Studies 1 and 2) can be found in Table 3. The other exact regression estimates are available within the sensitivity analysis document (see text footnote 2; for their summary see Supplementary Table 2).

**TABLE 3:** Regression estimates for all 3 studies.

**Estimates**	**b**	**SE**	***P***	**β**	**BF_10_**	**Posterior**
**SES ∼**						
Income	0.17	0.02	<0.001	0.45	5e16	1
	**0.22**	**0.02**	**<0.001**	**0.59**	**7e30**	**1**
	*0.17*	*0.03*	<*0.001*	*0.52*	*5e12*	*1*
**NEG ∼**						
SES	−0.30	0.05	<0.001	−0.32	1e05	1
	−**0.26**	**0.04**	**<0.001**	−**0.30**	**9e06**	**1**
**Stress ∼**						
SES	−0.35	0.05	<0.001	−0.37	3e05	1
	−**0.28**	**0.05**	**<0.001**	−**0.32**	**1e07**	**1**
	−*0.22*	*0.08*	*0.008*	−*0.25*	*20.8*	*0.95*
**WM ∼**						
NEG	0.12	0.23	0.597	0.12	0.06	0.05
	**0.16**	**0.18**	**0.377**	**0.16**	**0.05**	**0.04**
Stress	−0.38	0.23	0.096	−0.39	0.05	0.05
	−**0.31**	**0.17**	**0.073**	−**0.32**	**0.07**	**0.07**
	−*0.15*	*0.09*	*0.189*	−*0.12*	*0.08*	*0.07*
**SC ∼**						
NEG	−0.44	0.15	0.003	−0.41	1.22	0.55
	−**0.31**	**0.16**	**0.060**	−**0.28**	**0.43**	**0.30**
Stress	−0.05	0.15	0.746	−0.05	0.05	0.05
	−**0.26**	**0.16**	**0.113**	−**0.24**	**0.05**	**0.05**
	−*0.58*	*0.10*	<*0.001*	−*0.51*	*4e4*	*1*
**CR ∼**						
WM	0.60	0.13	<0.001	0.53	1e04	1
	**0.19**	**0.06**	**0.002**	**0.18**	**2.40**	**0.71**
	*0.05*	*0.10*	*0.637*	*0.05*	*0.07*	*0.06*
SC	−0.15	0.07	0.024	−0.15	3.75	0.79
	**0.10**	**0.06**	**0.060**	**0.12**	**0.12**	**0.10**
	−*0.08*	*0.09*	*0.418*	−*0.09*	*0.28*	*0.22*
**TD 1 ∼**						
CR	0.15	0.05	0.003	0.18	0.06	0.06
	**0.12**	**0.05**	**0.016**	**0.13**	**0.31**	**0.24**
	*0.42*	*0.63*	*0.510*	*0.06*	*0.21*	*0.17*
SC	−0.02	0.05	0.633	−0.03	0.10	0.09
	**0.02**	**0.05**	**0.727**	**0.02**	**0.05**	**0.05**
	*0.15*	*0.45*	*0.736*	*0.02*	*0.06*	*0.06*
FL	0.27	0.06	<0.001	0.27	500	1
	**0.18**	**0.05**	**0.001**	**0.18**	**3.31**	**0.77**
	*0.24*	*0.62*	*0.697*	*0.03*	*0.07*	*0.07*
**Risk−R ∼**						
CR	0.04	0.05	0.377	0.05	0.07	0.07
	**0.07**	**0.05**	**0.141**	**0.07**	**0.15**	**0.13**
SC	0.05	0.05	0.329	0.05	0.07	0.06
	−**0.03**	**0.05**	**0.473**	−**0.04**	**0.14**	**0.12**
FL	0.14	0.05	0.010	0.14	1.89	0.65
	**0.04**	**0.05**	**0.440**	**0.04**	**0.05**	**0.04**
**Risk−L ∼**						
CR	−0.14	0.04	0.001	−0.17	0.24	0.19
	**0.01**	**0.05**	**0.845**	**0.01**	**0.05**	**0.05**
SC	0.00	0.05	0.959	0.00	0.05	0.05
	−**0.08**	**0.04**	**0.071**	−**0.09**	**0.05**	**0.05**
FL	−0.10	0.05	0.042	−0.10	0.05	0.05
	−**0.10**	**0.05**	**0.027**	−**0.10**	**0.23**	**0.19**
**TD 2 ∼**						
CR	−**0.20**	**0.04**	**<0.001**	−**0.21**	**0.21**	**0.17**
SC	**0.05**	**0.04**	**0.266**	**0.06**	**0.37**	**0.27**
FL	−**0.22**	**0.05**	**<0.001**	−**0.22**	**4.11**	**0.80**
**TD 3 ∼**						
CR	*0.06*	*0.12*	*0.607*	*0.04*	*0.07*	*0.07*
SC	−*0.09*	*0.10*	*0.364*	−*0.07*	*0.08*	*0.08*
FL	*0.49*	*0.12*	<*0.001*	*0.34*	*84.3*	*0.99*

*Estimates from Study 1 are written in regular font, estimates from Study 2 are in bold, estimates from Study 3 are in italics. Income, equivalized household income; SES, subjective perception of own socioeconomic situation (only the financial satisfaction scale in Study 3); NEG, negative affect; Stress, perceived stress; WM, working memory; SC, self-control; CR, cognitive reflection; TD 1, staircase model of time-discounting; TD 2, IMCQ measure of time-discounting; TD 3, time-discounting with a real monetary reward (ordered categorical); Risk-R, staircase model of risk preference when a reward is involved; Risk-L, staircase model of risk preference when a loss is involved; FL, financial literacy.*

In general, the following can be inferred: (1) 13 out of 18 standardized regression coefficients (between Study 1 and 2) and 9 out of 18 coefficients (between all studies) were successfully cross-validated with the biggest difference equal to ± 0.1. This suggests relatively high stability across the datasets. (2) The markedly biggest differences in regression coefficients between the datasets were observed in the cases of the effect of working memory on cognitive reflection control (Δβ = 0.48) and the effect of stress on self-control (Δβ = 0.46). (3) In general, the specific regression coefficients were small and only 3 paths had a BF10 greater than 3 in all studies. This suggests more than just anecdotal evidence in favor of the alternative hypothesis (Jeffreys, 1961; Lee and Wagenmakers, 2013). (4) It was also found that the hypothesized predictors of economic preferences have null effects when a hypothetical reward is involved. In the case of real monetary reward, financial literacy has a substantive effect on one’s preference (β = 0.34). (5) After the exclusion of financial literacy in Studies 1 and 2, the majority of regression parameters only changed negligibly (the differences were in the hundredths). The only markedly bigger changes were observed in the BFs of the estimates between cognitive reflection and time-discounting measures (BF10 = 0.06 and 0.31 → BF10 = 1.24 and 4.90; and BF10 = 0.21 → BF10 = 2.45 for the second measure, respectively). Given the fact that each model has been falsified, some of the regression estimates might be biased and need to be interpreted with caution.

### Summary of the Sensitivity Analysis

The different methods of defining the measurement models (i.e., modeling all the items that remained after CFA; modeling all the items; modeling the best items) yielded very similar results to those obtained by parceling in terms of model fit (see Supplementary Table 3) and the needs for respecification. Regardless of how the measurement models were constructed in Study 1 and 2, the only identified substantial modification of the structural model was to add a covariance between negative affect and stress. After this modification, the presence of financial literacy in the model appeared to be the most crucial issue. The stability of the regression coefficients varied depending on the specific measurement model with the biggest deviations being caused by selecting the best items. The most stable coefficients were found between economic preferences and their predictors; the range of the differences between (point) regression estimates varied between Δβ = −0.29 −0.29. On the contrary, the least stable regression coefficients were found between self-control and negative affect as well as self-control and stress with Δβ≈1.2 as the difference between the smallest and the greatest value. The conducted sensitivity analysis confirms that while the model is constantly falsified regardless of the measurement model, the observed relationships are rather unstable (perhaps with the exception of the relationships between the aspects of economic decision-making and its predictors) and should be interpreted with particular caution.

## Discussion

The aim of the paper was to assess the verisimilitude of the hypothesized poverty perpetuation model by testing it against data from three independent studies. The results indicate that the proposed model has not found the necessary support in the data. Furthermore, the specific regression estimates could be biased, and the sensitivity analysis has revealed that they depend on the selected measurement model to a great extent.

The complexity of the model allows the discussion of many points (for partial interpretation of the results please see Adamkovič, 2019). In this paper, a meta insight will be provided into the factors that are likely to have caused the negative results (i.e., the fact that the proposed model was falsified, as well as the regression coefficients mostly indicating weak relationships between the levels of the model). Despite the existing assumptions that a poorer economic situation leads to less rational economic decision-making (e.g., Griskevicius et al., 2011; Brown et al., 2015; Pepper and Nettle, 2017), the data have indicated that the binary relationship between economic situation and economic preferences is weak (point estimates for objective economic situation: *r* = −0.08 −0.22; point estimates for subjective perception of economic situation: *r* = −0.13 −0.26) and have no substantial significance. In reality, the observed estimates are very similar to the existing empirical evidence (for a summary see Adamkovič et al., 2018). This implies that the narrative suggesting that people in poverty have less rational economic behavior, such as an unwillingness to delay gratification (Spears, 2011), is not based on ample evidence.

Likewise, many of the other relationships in the model were of a much lower magnitude than had been expected. It had been expected that cognitive load would deteriorate working memory. A meta-analysis on this topic (Shields et al., 2016) revealed the very small effects of stress on working memory, which would likely get even smaller after performing a regression-based correction for publication bias (Carter et al., 2019). The effect of financial literacy on economic preferences can serve as another example. According to previous studies (e.g., Gathergood, 2012; Lusardi et al., 2017; Grohmann, 2018), it could be expected that financial literacy would determine economic preferences. Counterintuitively, a meta-analysis by Fernandes et al. (2014) suggests that an increase in financial literacy explains less than 1% of the variance in economic decision-making. Furthermore, after controlling for variables (such as one’s willingness to take risks or tendency to plan), financial literacy ceases to be a significant predictor of economic preferences. In this case, it seems that factors determining economic preferences go beyond the construct of financial literacy and could be of a more general nature [in other words, the perception of time (Zimbardo et al., 2017) or level of education (Kim et al., 2018)]. These two examples indicate that some of the hypothesized relationships might have been based on weak, or perhaps even incorrect, assumptions. As discussed in the limitations of the study, constructing a complex theory by synthesizing the existing evidence could be a tricky task, especially given the replicability issues that behavioral sciences have to face.

The hypothesized cognitive mechanism does not hold true as a whole. It shows mostly weak relationships between the variables and indicates that the facets of economic decision-making are virtually unrelated to any other variables. This raises the question as to what variable or variables determine one’s economic preferences. One explanation might dwell in a shift of the perspective. It is worth considering if economic preferences are stable autonomous (personality) traits (Odum, 2011; Peysakhovich et al., 2014; Meier and Sprenger, 2015; Frey et al., 2017; Pedroni et al., 2017) substantively determined by relatively urgent needs or situations of a financial (Bickel et al., 2016) or emotional (Schildberg-Hörisch, 2018) nature, rather than by one’s abilities or knowledge. In order to test this assumption, the stability of the specific economic preferences in time was calculated on a sample of 224 participants who had completed the test battery twice. The results revealed moderately strong correlations (Pearson’s *r*) of preferences in time (the time difference between the data collections was approximately 1 year): time-discounting = 0.41, 95% CI (0.28, 0.52); risk-reward = 0.42, 95% CI (0.29, 0.53); and risk-loss = 0.44, 95% CI (0.29, 0.56). However, the trait-like approach to economic preferences might have its flaws. Previous studies which examined the time stability of this sort (Odum, 2011; Chuang and Schechter, 2015; Schildberg-Hörisch, 2018) reported various stabilities for different time intervals. In general, the evidence shows a predictable pattern: the shorter the time interval between the measures, the higher the correlation.

Besides the psychological interpretations, the results could be explained by the core sociodemographic characteristics of the research samples. The recent OECD reports (OECD, 2018a, OECD, 2018b) showed that Slovakia has the lowest inequality rate (GINI coefficient = 0.24) and one of the lowest poverty rates (8.5%) out of the surveyed countries. These macroeconomic circumstances allow the majority of people to make ends meet. Once their basic material needs are met, economic decisions could be indeed determined by one’s preferences in general. This is irrelevant of objective financial situation, financial literacy, or the whole cognitive mechanism. This would provide even more support for the argument that intrinsic economic preferences are, to a great extent, autonomous behavioral patterns similar to the common comprehension of personality traits.

If poverty perpetuation cannot be explained by this mechanism, it is of interest to identify the prototype of a person whose economic preferences are more myopic. Beyond the main scope of the study, it was decided to calculate a latent class analysis in order to examine this. Despite the number of extracted classes (2–6), it was not possible to find any meaningful categorization. In other words, people within the classes were so distinct that there was no theoretically justifiable, and even less predictable, pattern of characteristics. If poverty perpetuation indeed depends on one’s economic decision-making rather than general (e.g., level of education) or external (e.g., “inherited” economic status) factors, it is necessary to explain and empirically examine the parts of this process. Yet, it is unknown what alternative mechanism (see, for example, instance-based learning theory; Gonzalez et al., 2003; Gonzalez, 2013) can appropriately explain or help to predict the relationship between economic situation and economic preferences.

### Limitations and Directions for Future Research

The attempt to assess the verisimilitude of such a complex model has several potential pitfalls. The most important of these have been selected for discussion. These limitations are not necessarily exclusive to the conducted studies and can occur in future research on this topic or on similar ones. Since the majority of them are of a systematic nature, how they can be handled/not handled in future studies is discussed. For this reason, general respecifications of the hypothesized model are not suggested (i.e., there are too many ways the parts of the model could be plausibly updated). Rather, each limitation is elaborated on and some ideas are offered as to how they can be addressed, and new directions for future research are provided.

(1) As previously discussed, the first issue is related to researcher degrees of freedom. The selection of the most appropriate variables and forming them into a unitary model while taking the most plausible flow of causality between the variables into account is not really straightforward and can have numerous alternatives. At the same time, psychological phenomena are often so complex and complicated that a researcher has to compromise between capturing all the potentially relevant variables and parsimony of the model. Thus, testing this exact model is not the only option in capturing poverty perpetuation at the level of an individual, and future research on the topic could try to cover it very differently.
(2) Based on existing research, it can be assumed that cognitive load is related to both poverty and decision-making (Schilbach et al., 2016; Capraro, 2019). However, its operationalization might be problematic. According to the definition, cognitive load indicates the presence of a burden on the cognitive system that diminishes one’s ability to focus on different stimuli (Paas and Van Merriënboer, 1994; Sweller et al., 1998). In behavioral research, researchers almost always use experimental tasks (e.g., memory tasks or the Stroop task; see, for example, Deck and Jahedi, 2015; Janssen et al., 2019) to induce cognitive load. In the context of a long-term condition, such as poverty, researchers could assess cognitive load either indirectly (e.g., via related factors such as experiencing negative affect or stress) or directly. The direct approach might lean on the assumption that people are self-aware of how much mental effort they invest in thinking about their current economic situation. The construction and validation of a measure of cognitive load related to the financial situation (similar to the Mental-effort rating scale; Paas, 1992) could be another important step in having a better insight into the process of poverty perpetuation.
(3) In all three studies, the CRT was used (Frederick, 2005) coupled with three additional items created by Oldrati et al. (2016) as a measure of intuitive/deliberative thinking. In Study 3, the obtained parameters suggested that removing cognitive reflection from the model would markedly increase its fit. Firstly, this was considered to be a statistical artifact, but after further exploration of the data from Studies 1 and 2, it was found that the exclusion of this variable markedly improved the model fit. As this finding indeed contradicts the theoretical assumptions, two explanations are provided as to why this might have happened. Firstly, there is some evidence (Pennycook et al., 2016) that the CRT might be a valid measure of deliberative, but not of intuitive, thinking. The lack of deliberation is likely not the same as relying on intuition. A possible alternative for future research would be employing a different measure (or perhaps a pair of measures) that can validly assess the extent of one‘s intuitive/reflective thinking. Secondly, the relationship between cognitive reflection and time/risk preference has been weak throughout both the primarily reported analyses and the sensitivity analysis. With the initial assumption that the score obtained from the CRT measure is valid, this finding supports the hypothesis that both time and risk preferences are autonomous traits, practically independent of one‘s cognitive abilities or tendencies.
(4) Another possible limitation can be the study of economic preferences on hypothetical rewards. The current empirical evidence which examines whether decision-making involving hypothetical rewards is the equivalent of decision-making with real financial incentives [for a summary, see Xu et al. (2016)] has brought ambiguous results. The current data in this paper suggests that the model does not hold true even after incentivizing participants with real monetary rewards. Moreover, the binary relationships between the indicators of economic status and economic preference are still weak. One of the directions for future research could focus on using more realistic scenarios (e.g., specific consumer behavior, taking and managing loans or gambling) as the indicators of economic decision-making. The preference does not necessarily equal the actual decision. For instance, a person facing material hardship could indeed express a genuine willingness to delay the gratification. However, in a situation with a financial reward (given the equivalent amount of money), it might be much easier to succumb to the vision of having the money “here and now”. Unfortunately, it is very challenging to conduct a study in the current research world of behavioral science with a sufficiently powered design where all participants are incentivized with relatively high payments (that are similar to the hypothesized scenarios). An increase in incentives will be associated with a magnitude effect (people are more willing to delay gratification as the value of the reward increases; see Ballard et al., 2017), potentially leading to different outcomes.
(5) In the proposed model, the time and risk preferences were related to situations that involved financial decision-making. It was believed that this would have provided the most straightforward illustration of why people fall into the poverty trap. Given that the model was disconfirmed and that the observed correlations between the indicators of economic situation and these preferences were weak, this indicates that there may well be other aspects of decision-making that contribute to poverty perpetuation. As a result of the social aspects associated with poverty (see Mood and Jonsson, 2015), future research on this topic could tackle social, or perhaps even moral (see, for example, Fehr and Schmidt, 1999; Andreoni et al., 2017; Capraro and Rand, 2018), preferences. It is possible that the consequences of poverty (such as experiencing negative affect, stress, or impaired cognitive functions) negatively impact many aspects of one‘s social life (e.g., causing problems in maintaining one‘s social network in terms of frequency of meetings and quality of the relationships; disregarding the importance of asking for/accepting help; lowering one’s willingness to participate in public/organizational activities; etc.). The neglect of these opportunities might be coupled with apathy and the loss of motivation to change the situation. Therefore, more research is needed to distinguish to what extent poverty perpetuation is driven by suboptimal social rather than suboptimal financial decisions.
(6) In terms of the methodological aspects of this research, analyzing only observational data may be an issue that could have contributed toward the disconfirmation of the model. However, it is believed that such a complex problem could not be tackled with an experimental approach. Hence, analyzing the data obtained by self-reported measures (as is standard in psychology) is seen to be a more appropriate way to answer the given research question, even after admitting its caveats.
(7) The falsification of the model can also be attributed to problems of a systemic nature, such as publication bias (e.g., Fanelli, 2012) or a lack of good theory (e.g., Muthukrishna and Henrich, 2019) in behavioral sciences. Indeed, at least some of the theories upon which the model has been built might be flawed and, hence, integrating these non-valid assumptions into one holistic model should bring negative results by construction. The currently pronounced replicability crisis (for example, Open Science Collaboration, 2015; Camerer et al., 2018) has already led to substantial improvements in practices and psychology’s renaissance (Nelson et al., 2018). Therefore, independent researchers are invited to conduct replications on different (non-WEIRD) samples and under different settings (e.g., incentives, measures, etc.), even with modified versions of the hypothesized model.

## Conclusion

Haushofer and Fehr (2014) emphasized the need to study the factors which potentially contribute to poverty perpetuation. Adamkovič (2019) proposed a theoretical model of a cognitive mechanism, describing how poverty affects one’s economic preferences via a cognitive mechanism. Based on the data from 3 studies, it can be concluded that the model was falsified, and the hypothesized mechanism does not explain why a person is trapped in the poverty cycle. Furthermore, even the bivariate relationships between the variables are much smaller than had been expected. The conducted sensitivity analysis has revealed that the majority of the obtained estimates are stable across the datasets, although they vary substantively due to arbitrary decisions related to the conceptualization of the measurement models. There are 3 main reasons which were identified as to why the model did not hold true: (1) Economic preferences have characteristics of stable traits and thus are only weakly determined by one’s cognitive abilities or persistent mental load. Instead, they could be subjected to (relatively urgent) needs that are caused by contextual factors. (2) The relatively high level of social equality and relatively low poverty rate in Slovakia implies that the majority of its inhabitants can afford the most necessary commodities and meet their essential needs regardless of their actual economic situation. Therefore, economic preferences might be independent of financial situation. (3) The body of knowledge in behavioral sciences suffers from several problems (e.g., lack of good theory, ambiguous and vague conceptualizations of constructs, the complicated issue of causality, questionable research practices, publication bias), leading to low replicability, and hence credibility of the current findings. This indicates that the theories used to conceive the model might not hold true after a more thorough examination, and thus the model is flawed in its construction. Even though the discussion and conclusions presented in this paper come from 3 different studies, it is important to highlight the need for further replication under different conditions and with enhanced models. For knowledge to be credible, robust evidence is needed.

## Data Availability Statement

Code and data are available at https://osf.io/qy8mn/ and in Supplementary Data Sheet 1.

## Ethics Statement

The studies involving human participants were reviewed and approved by the Etická komisia (ethics board) at the Institute of Psychology, Faculty of Arts, University of Prešov. The participants provided their written informed consent to participate in this study.

## Author Contributions

MA was a co-author of the theoretical framework of the tested model, designed the study, performed the analyses, and discussed the findings.

## Conflict of Interest

The author declares that the research was conducted in the absence of any commercial or financial relationships that could be construed as a potential conflict of interest.

## Footnotes

1 https://osf.io/z3jyf

2 https://osf.io/r97mt/
