# Supplementary material for: Consequences of Poverty on Economic Decision-Making: Assessing the Verisimilitude of the Cognitive Mechanism
Source: Front Psychol. 2020 Feb 13;11:171. doi: 10.3389/fpsyg.2020.00171 (PMC7031509; doi:10.3389/fpsyg.2020.00171)
Supplement: Supplementary file 2 [file Table_1.PDF]

Supplementary Table 1. CFAs for all 3 studies

Measure	χ^2	df	p	CFI	TLI	RMSEA [95% CI]	SRMR
Study 1							
NEG	335.38	22	< .001	.85	.94	.19 [.17, .21]	.07
NEG (mod)	29.29	8	< .001	.99	.99	.08 [.04, .13]	.02
Stress	436.87	13	< .001	.75	.79	.28 [.26, .30]	.12
Stress (mod)	23.48	8	.003	.99	.99	.07 [.02, .12]	.02
WM	30.66	23	.131	.97	.97	.03 [.00, .05]	.09
WM (mod)	10.67	12	.557	1	1	.00 [.00, .05]	.05
SC	272.52	42	< .001	.78	.87	.12 [.10, .13]	.07
SC (mod)	31.08	17	.020	.98	.99	.05 [.00, .08]	.03
CR	10.28	8	.246	1	1	.03 [.00, .07]	.04
FL	11.65	8	.168	.99	.99	.03 [.00, .08]	.05
Study 2							
NEG	315.14	22	< .001	.89	.96	.16 [.14, .18]	.07
NEG (mod)	73.47	8	< .001	.98	.98	.13 [.10, .17]	.03
Stress	458.59	20	< .001	.81	.90	.21 [.19, .23]	.08
Stress (mod)	56.33	8	< .001	.98	.99	.12 [.07, .16]	.03
WM	75.76	17	< .001	.96	.96	.09 [.07, .10]	.11
WM (mod)	14.81	7	.039	1	.99	.05 [.00, .09]	.05
SC	287.16	41	< .001	.80	.85	.11 [.10, .12]	.07
SC (mod)	94.36	16	< .001	.89	.90	.10 [.08, .12]	.05
CR	19.73	8	.011	.99	.99	.06 [.02, .09]	.04
FL	34.65	18	.010	.97	.96	.04 [.02, .07]	.06
FL (mod)	29.63	13	.005	.97	.96	.05 [.02, .08]	.06
Study 3							
Stress	135.01	21	< .001	.88	.92	.15 [.13, .18]	.07
Stress (mod)	18.86	7	.009	.99	.98	.08 [.03, .15]	.03
WM	57.80	10.29	< .001	.87	.84	.14 [.10, .18]	.14
WM (mod)	8.00	2.67	.035	.98	.96	.09 [.00, .20]	.05
SC	201.05	33	< .001	.66	.68	.15 [.13, .16]	.10
SC (mod)	65.73	15	< .001	.86	.84	.12 [.09, .15]	.07
CR	6.38	8	.605	1	1.01	.00 [.00, .07]	.05
FL	23.89	17	.123	.92	.90	.04 [.00, .08]	.08
FL (mod)	14.29	13	.354	.99	.98	.02 [.00, .07]	.07

Note: (mod) = denotes a modified version of the scale (based on the exploratory analysis); NEG = negative affect; Stress = perceived stress; WM = working memory; SC = self-control; CR = cognitive reflection; FL = financial literacy

Sensitivity analysis

Below you can find the summary of the results of the regression estimates and model fits obtained by the sensitivity analysis based on the different measurement models for all 3 studies (24 estimated models in total). For the specific results please see https://osf.io/r97mt/

Supplementary Table 2. Summary of the standardized regression coefficients (β) obtained by the sensitivity analysis

Regressions:	Min	Max	Range
subjPoverty ~			
houseInc	.21	.60	.39
NEG ~			
subjPoverty	-.94	-.27	.67
STRESS ~			
subjPoverty	-.98	-.24	.74
WM ~			
NEG	-.18	.54	.72
STRESS	-.65	-.08	.57
SELFC ~			
NEG	-.55	.72	1.27
STRESS	-1.20	-.03	1.17
CR ~			
WM	-.07	.61	.68
SELFC	-.18	.32	.50
TD 1 ~			
CR	.08	.23	.15
SELFC	-.04	.05	.09
FL	.13	.29	.16
RR ~			
CR	-.01	.08	.09
SELFC	-.06	.11	.17
FL	-.01	.15	.16
RL ~			
CR	-.21	.08	.29
SELFC	-.13	.01	.14
FL	-.11	.00	.11
TD 2 ~			
CR	-.29	-.14	.15
SELFC	.03	.13	.10
FL	-.22	-.15	.07
TD 3 ~			
CR	.10	.18	.08
SELFC	.03	.09	.06
FL	.52	.55	.03

Note: NEG = negative affect; STRESS = perceived stress; WM = working memory; SELFC = self-control; CR = cognitive reflection; TD 1 = staircase model of time-discounting; TD 2 = IMCQ measure of time-discounting; TD 3 = time-discounting with a real monetary reward (ordered categorical); Risk-R = staircase model of risk preference when a reward is involved; Risk-L = staircase model of risk preference when a loss is involved; FL = financial literacy

Supplementary Table 3. Summary of the chi-square statistics and AFI obtained by the sensitivity analysis

Model	χ^2	df	p	CFI	TLI	RMSEA [95% CI]	SRMR
**CFA-based items**							
Study 1							
Initial	839.95	169	< .001	.81	.86	.10 [.09, .10]	.11
W/ cov.	535.10	169	< .001	.90	.92	.07 [.07, .08]	.10
W/o FL	355.89	155	< .001	.95	.96	.06 [.05, .06]	.07
Study 2							
Initial	1051.63	189	< .001	.82	.87	.10 [.09, .10]	.10
W/ cov.	717.12	189	< .001	.89	.92	.08 [.07, .08]	.10
W/o FL	532.04	170	< .001	.92	.95	.07 [.06, .07]	.08
Study 3							
Initial	304.87	124.34	< .001	.75	.77	.08 [.07, .08]	.13
W/o FL	263.39	105.92	< .001	.81	.83	.08 [.07, .09]	.11
**All items**							
Study 1							
Initial	906.26	207	< .001	.80	.85	.09 [.09, .09]	.10
W/ cov.	690.01	207	< .001	.86	.90	.08 [.07, .08]	.10
W/o FL	592.29	195	< .001	.89	.92	.07 [.07, .07]	.08
Study 2							
Initial	988.56	221	< .001	.84	.89	.09 [.08, .09]	.08
W/ cov.	682.44	219	< .001	.90	.93	.07 [.06, .07]	.07
W/o FL	677.79	213	< .001	.90	.93	.07 [.07, .07]	.08
Study 3							
Initial	346.92	146.52	< .001	.75	.77	.08 [.07, .08]	.12
W/o FL	323.85	129.87	< .001	.79	.81	.08 [.07, .08]	.11
**Best items**							
Initial	911.34	110	< .001	.78	.82	.13 [.13, .14]	.13
W/ cov.	458.41	110	< .001	.90	.92	.09 [.08, .09]	.11
W/o FL	255.02	98	< .001	.96	.97	.06 [.06, .07]	.07
Study 2							
Initial	961.17	119	< .001	.81	.85	.12 [.12, .13]	.11
W/ cov.	477.78	118	< .001	.91	.93	.08 [.08, .08]	.09
W/o FL	349.62	104	< .001	.94	.96	.07 [.06, .08]	.07
Study 3							
Initial	248.54	88.79	< .001	.74	.76	.09 [.08, .09]	.13
W/o FL	191.12	72.66	< .001	.81	.83	.08 [.07, .09]	.11

Note: Initial = the initial conceptualization of the model; W/ cov = the model with a covariance term between negative affect and stress; W/o FL = the model without financial literacy
